# Behavioral Therapy Across the Spectrum

**Published:** 2011

**Authors:** Katie Witkiewitz, G. Alan Marlatt

**Keywords:** Alcohol use disorders (AUDs), alcohol and other drug use (AODU) treatment method, behavioral therapy, individual therapy, facilitated self-change, family or couples therapy, contingency management, cognitive–behavioral therapy, efficacy

## Abstract

Numerous effective behavioral therapies have been developed that can bring the treatment to the patient rather than bringing the patient to treatment. These behavioral therapy techniques, which can provide effective treatment across the spectrum of severity of alcohol abuse disorders, include facilitated self-change, individual therapies, couples and family approaches, and contingency management. New methods of delivery and successful adjuncts to existing behavioral treatments also have been introduced, including computerized cognitive–behavioral treatments, Web-based guided self-change, and mindfulness-based approaches. Although a wide variety of behavioral approaches have been shown to have good efficacy, choosing the treatment most appropriate for a given patient remains a challenge.

Since the mid-1980s and 1990s, behavioral treatment of alcohol abuse and dependence (i.e., alcohol use disorders [AUDs]) has advanced steadily. This article introduces different types of behavioral treatment, summarizes the evidence for their efficacy, and describes alternative methods of delivery and adjuncts to existing treatments that might appeal to some patients. In addition, the article discusses the importance of moving beyond a focus on comparing the effectiveness of existing active behavioral treatments and toward a research agenda that considers more thoughtfully how people change as well as the mechanisms of change during the course of behavioral treatments.

## Behavioral Treatment Approaches

Several distinct treatments exist under the general rubric of behavioral treatments for AUDs, including coping skills training, relapse prevention and other cognitive–behavioral treatments, contingency management approaches, brief behavioral interventions, behavioral couples and family treatments, facilitated self-change approaches, and aversion therapy. Many other alcohol treatments also incorporate behavioral principles. For example, 12-step groups (e.g., Alcoholics Anonymous) often rely on positive reinforcement (e.g., by recognizing abstinence anniversaries) and behavioral modeling (e.g., by having a sponsor). Motivational interviewing (see Miller and Rose 2009) also often relies on behavioral principles (e.g., reinforcement, modeling) within the treatment session. This review highlights those interventions that are rooted in behavior therapy (see table). All of these treatments can be delivered in individual sessions or group formats, and many of them have been adapted to be delivered in a variety of treatment settings, including residential, outpatient, computerized, medical, and workplace settings.

### Conceptual Overview

All behavioral approaches to treatment of AUDs combine an attention to general behavioral principles (e.g., reinforcement and punishment) with therapeutic techniques designed to facilitate healthy behavior change. Coping skills training, cognitive behavioral treatment, brief behavioral interventions, and relapse prevention also introduce concepts from cognitive therapy and social learning theory, primarily the identification of cognitions related to alcohol use and situations in which maintaining abstinence might be challenged. For example, the cognitive concept of self-efficacy, or belief in one’s ability to abstain from alcohol, plays a prominent role in both cognitive–behavioral treatments and relapse prevention. Likewise, an individual’s expectations regarding the effects of alcohol (i.e., expectancies) often are identified and challenged during the course of cognitive–behavioral interventions. Coping skills training and relapse prevention primarily focus on identifying high-risk situations for drinking and then building a repertoire of coping skills to help patients approach risky situations without using alcohol. Brief interventions, such as brief physician advice (Fleming et al. 2000) and the Brief Alcohol Screening and Intervention for College Students (BASICS) approach (Dimeff et al. 1999), also utilize many cognitive–behavioral tools; however, in these cases, treatment occurs over a short period of time (often an hour or less). The effectiveness of these approaches has been demonstrated in numerous studies. For example, Fleming and colleagues (2000) found that brief physician advice, delivered across two physician visits and two follow-up phone calls, resulted in a significant reduction in alcohol use and binge-drinking episodes for up to 4 years following the intervention. More recently, a study found that brief interventions were equally effective for alcohol-dependent and nondependent participants (Guth et al. 2008).

#### Contingency Management Approaches

Contingency management approaches rely more exclusively on the principles of operant conditioning—that is, they use reinforcing and punishing consequences to maintain positive behavior change. Contingency management approaches, which often are used as an adjunct to another treatment, share three central components:

- Monitoring the individual carefully (e.g., using urinalysis or blood tests) so that alcohol use is identified;
- Providing tangible positive rewards (such as vouchers that can be exchanged for retail goods or cash) for a desired behavior (e.g., abstinence from alcohol); and
- Withholding rewards (e.g., vouchers) or implementing other negative consequences (e.g., providing negative reports to interested other parties, such as family members or parole officers) when alcohol use is identified.

Cognitive therapy typically is not part of a contingency management treatment; however, contingency management can lead to increased self-efficacy for abstinence (Litt et al. 2009), potentially by providing individuals with the experience of being abstinent from alcohol (Witkiewitz and Marlatt 2008).

#### Behavioral Couples, Marital, and Family Therapy

These approaches incorporate a thorough assessment of drinking behaviors and an analysis of relationship factors that may influence these behaviors, including communication, conflicts, and problem solving. Both behavioral couples treatment (McCrady and Epstein 1995) and marital family therapy (O’Farrell et al. 1993) incorporate several behavioral techniques designed to reduce drinking and drinking-related problems as well as increase caring behaviors, enhance communication, and improve relationship functioning. Recent studies have found that both behavioral couples therapy and behavioral family therapy are related to better outcomes following treatment than behavioral individual therapies (see McCrady et al. 2009; O’Farrell et al. 2010). Skills training, contingency management, and behavioral contracting often are primary components of these treatments.

#### Facilitated Self-Change

The majority of people with AUDs do not seek treatment, and most of them are able to quit drinking or maintain moderate drinking without receiving formal treatment. Thus, most people quit drinking on their own. In light of these findings, several treatments have been developed that aim to facilitate self-change. For example, behavioral self-control training (Miller and Munoz 1982) and guided self-change (Sobell and Sobell 1993; also see Klingemann and Sobell 2007) are two programs that have received considerable empirical support for reducing alcohol use and alcohol-related problems. For most facilitated self-change programs, primary treatment goals include goal setting, self-monitoring of drinking behavior, analysis of drinking situations, and learning alternate coping skills. Many of these treatment approaches are delivered via self-help workbooks or computer programs, are Internet based (e.g., Smart Recovery), or are administered via mailed interventions. Facilitated self-change approaches also can be therapist directed in individual or group formats.

#### Aversion Therapy

Aversion therapy relies almost exclusively on behavioral principles of conditioning. The goal is to help patients reduce or eliminate their alcohol use behavior by conditioning a negative response (e.g., an electric shock or nausea) to cues that were previously associated with drinking. In some cases, such as treatment with the drug disulfiram (Antabuse^®^), patients will have a highly unpleasant physical reaction if they consume even small amounts of alcohol.^1^ Imagining unpleasant scenes combined with imagery of drinking (i.e., covert sensitization) also has been used as a form of aversion therapy (Rimmele et al. 1995). In general, however, aversion therapies are not widely used today.

### Efficacy of Behavioral Treatments

Several reviews and meta-analyses of the research literature have determined that behavioral treatments—including brief intervention, marital and family therapy, behavioral couples therapy, relapse prevention, and other cognitive– behavioral treatments as well as community reinforcement and contingency management approaches—are among the most effective treatments for AUDs (see Finney and Monahan 1996; Miller and Wilbourne 2002). Specifically, study findings included the following:

- Recent meta-analyses of cognitive– behavioral treatments (Magill and Ray 2009) and contingency management approaches (Prendergast et al. 2006) have concluded that effect sizes for either treatment approach range from small to medium, depending on the comparison group (e.g., active treatment or control group), definition of outcome (e.g., abstinence or reduced alcohol problems), and follow-up time (e.g., 6 vs. 12 months after treatment).
- A meta-analysis of behavioral couples, marital, and family therapy (Powers et al. 2008) found that for married or cohabiting patients, these approaches yielded medium to large effects and better outcomes than individual-based treatments.
- A meta-analysis of 17 studies evaluating behavioral self-control training (BSCT) indicated that this approach produced moderately strong effects in comparison to no intervention and smaller effects in comparison to abstinence-oriented comparison treatments (Walters et al. 2000).

To more accurately compare the effectiveness of treatments across different studies using different study designs, Miller and Wilbourne (2002) created a cumulative evidence score that takes into account the treatment effects as well as the methodological strengths and weaknesses of the studies. This score was used to ascertain the effectiveness of different treatments based on 361 controlled studies. Of the psychosocial interventions analyzed, brief interventions had the highest cumulative evidence, yielding significant reductions in drinking across most studies, even in non–treatment-seeking populations. Behavioral interventions, including community reinforcement, behavioral contracting, behavioral marital therapy, skills training, chemical aversion therapy, covert sensitization, and self-control training, also ranked in the top 20 of all treatment modalities (Miller and Wilbourne 2002). In addition, relapse prevention, contingency management, Drinker’s Check-up, and behavioral couples’ therapy have been identified as effective by the Substance Abuse and Mental Health Services Administration (SAMHSA) National Registry of Evidence-Based Programs and Practices (see www.nrepp.samhsa.gov).

However, although many behavioral treatments have been found to be effective, a recent meta-analysis has questioned whether these various behavioral treatments result in significantly different outcomes compared with other bona fide psychological treatments^2^ for AUDs (Imel et al. 2008). In a review of 30 studies that had compared at least two bona fide psychotherapies, these investigators found that net effect sizes across treatments were not significantly different from zero, suggesting that all treatments produced similar effects. Looking at individual studies, the investigators also found that authors’ allegiance to a particular treatment explained a significant portion of the variability between different treatment outcomes. In addition to these findings, there is scant evidence to support the efficacy of these behavioral treatments with minority groups and among patients with comorbid mental health disorders, and future meta-analyses are desperately needed to determine which treatments work best for these groups.

### Adaptations of Existing Behavioral Treatments

Alcoholism treatment can be provided in a wide range of settings. Several outcome studies have concluded that inpatient (i.e., residential) treatment offers no advantages over outpatient treatment of alcohol dependence. Also, research on alcohol screening and intervention in primary-care facilities (Fleming et al. 2000) and emergency departments or trauma centers (Gentilello et al. 1995; Monti et al. 2007) indicates that these alternative treatment settings might be essential for helping people who otherwise would have not sought treatment. Accordingly, those treatment approaches that can be adapted to different treatment settings are particularly useful. Most of the behavioral approaches described above can be adapted for multiple settings (e.g., inpatient or outpatient treatment, community centers, schools, primary-care clinics, or emergency rooms) and delivery methods (e.g., phone, Internet, computer-based, postal mail), and a growing body of research evidence supports the adaptability of behavioral interventions. The adaptation of these approaches to different delivery methods, in particular, has great promise to change the face of treatment for AUDs. As discussed in more detail in the article by Gustafson and colleagues (pp. 327–337 in this issue), computer and Web-based approaches are likely to greatly expand the availability of evidence-based behavioral treatment strategies. For example, an approach called Computer-Based Training in CBT (CBT4CBT) can predict greater treatment engagement and decreased drug use compared with usual treatment (Carroll et al. 2008). Similarly, the Drinker’s Check-up, a computer-based brief intervention, can reduce the quantity and frequency of drinking by 50 percent, with reductions sustained through 12 months following the intervention (Hester et al. 2005). Finally, the National Institute on Alcohol Abuse and Alcoholism (NIAAA) recently launched a self-change Web site and booklet called *Rethinking Drinking* (http://rethinkingdrinking.niaaa.nih.gov/) that provides interactive feedback and tools for helping people cut back on their drinking. *Rethinking Drinking* is freely available and has the ability to reach millions of people who might be thinking about changing their drinking behavior on their own.

In another adaptation of existing treatments, recent research indicates the potential value of adding mindfulness training to existing behavioral treatments for AUDs. Relapse prevention, which best can be characterized as a cognitive–behavioral approach focusing on coping skills training and identification of high-risk situations for relapse, has been expanded to incorporate 8 weeks of group training in mindfulness meditation (Bowen et al., in press). The results suggest significant reductions in substance use, including alcohol use and polysubstance use, and craving for substances in the first four months following the intervention (Bowen et al. 2009).

## Understanding How People Change

The majority of meta-analyses and controlled treatment trials have concluded that most active treatments are equally effective; therefore, it might be more important to focus on defining exactly what treatment components are responsible for this effectiveness. For example, Moos (2007) described four related theories that help explain the active ingredients that are common to most effective treatments, drawing upon social control theory, behavioral economics and behavioral choice theory, social learning theory, and stress and coping theory to explain common components of effective treatment. According to this analysis, important components included the following:

- Social support;
- Structure and goal direction;
- Provision of rewards and rewarding of activities;
- Normative models for successful abstinence;
- Enhancement of self-efficacy; and
- Teaching of coping skills.

A focus on such empirically supported treatment processes, rather than on different treatment modalities, might provide an opportunity for a more general treatment of AUDs that is linked explicitly to the core processes which instigate and maintain problematic drinking patterns.

Those in the alcohol research field have learned over the years that many people change between making the decision to enter treatment or an initial evaluation and actually starting the first treatment session. Consistent with this observation, the provision of specific treatments targeted to address certain individual characteristics determined at pretreatment evaluation has not led to substantial improvements in treatment outcomes (Project MATCH Research Group 1998). For example, recent analyses of data from the COMBINE Study (COMBINE Study Research Group 2006)^3^ indicated that craving scores decreased significantly between the baseline assessment and the first treatment session (Witkiewitz 2009). Likewise, people with lower levels of craving at baseline did not especially benefit from receiving a specialized treatment session designed to impact craving. Thus, identifying a specific treatment for a certain person (e.g., motivation enhancement therapy for a person with low motivation) may be less useful than identifying those treatment elements and settings that are most appropriate for a given patient. For example, a patient with no social support system potentially might receive greater benefit from a behavioral treatment that provided social support or skills for increasing social support for abstinence. Conversely, a person who does not have much time to attend treatment sessions might benefit more from a Web-based intervention. And people who are concerned about the implications of receiving formal treatment might be best suited by self-change methods. Thus, it is important for treatment professionals, concerned family members, and patients who want to change their drinking behavior to consider “what will work best for me?” rather than “what treatment works best?” For researchers it is imperative to devote more attention to evaluating what treatment processes, settings, and delivery methods work best for which patient and how people change their drinking behavior over time.

It also is critical to note that many studies consistently find reductions in alcohol use among control groups who do not receive behavioral treatments (e.g., Weiss et al. 2008) and among people who do not seek formal treatment (Sobell et al. 2000; Tucker et al. 2004), and it would be useful to understand the mechanisms contributing to these changes. Furthermore, few treatment studies to date have examined long-term outcomes and often do not report on morbidity, mortality, or costs of persistent alcohol use following treatment.

## Conclusions

As the studies reviewed here indicate, a wide array of behavioral treatments for AUDs produce significant reductions in alcohol use and alcohol-related problems. People who seek to reduce their alcohol use or quit drinking now are presented with a plethora of options and opportunities for changing their drinking behavior without needing to check in for a 28-day inpatient hospitalization, attend Alcoholics Anonymous meetings on a daily basis, or commit to an abstinence goal. The behavioral approaches described in this article share many treatment processes and are generally based on the same underlying theories of behavior. Therefore, investigations focusing on selecting those treatment processes, settings, and delivery methods that most suit the specific needs of a given patient are a fruitful area of future inquiry.

## Figures and Tables

**Table:** Overview of Behavioral Therapies

**Therapy**	**Primary Focus**	**Manual (if available) and Other Resources**
**Coping skills training**	Social learning theory and skills training to enhance individual coping skills. Also includes cue exposure with response prevention to extinguish association between alcohol cues and alcohol seeking.	Monti, P.M.; Kadden, R.M.; Rohsenow, D.J.; et al. *Treating Alcohol Dependence: A Coping Skills Training Guide.* Second Edition. New York: Guilford Press, 2002. Kadden, R.M. *Cognitive-Behavior Therapy for Substance Dependence: Coping Skills Training,* 2002. Available at: http://www.bhrm.org/guidelines/CBT-Kadden.pdf
**Relapse prevention and cognitive–behavioral therapy**	Identifying client’s high-risk situations for relapse and using cognitive and behavioral techniques to help clients cope with risky situations.	Daley, D.C., and Marlatt, G.A. 2006. *Overcoming Your Alcohol or Drug Problem: Effective Recovery Strategies Workbook.* New York: Oxford University Press, 2006. Marlatt, G.A.and Gordon, J.R. (Eds.). *Relapse Prevention. Strategies in the Treatment of Addictive Behaviors.* New York: Guilford Press, 1985. Marlatt, G.A.; Parks, G.A.; and Witkiewitz, K. *Clinical Guidelines for Implementing Relapse Prevention Therapy,* 2002. Available at: http://www.bhrm.org/guidelines/RPT%20guideline.pdf
**Contingency management**	Using reinforcing and punishing consequences to alter substance use behavior. Requires identification of client-specific consequences and making receipt of consequences contigent on some desired behavior (e.g., abstinence).	Higgins, S.T.; Silverman, K.; and Heil, S.H. (Eds.). *Contingency Management in Substance Abuse Treatment*. New York: Guilford Press, 2008. Petry, N.M. *A Clinician’s Guide for Implementing Contingency Management Programs,* 2001. Available at: http://www.bhrm.org/guidelines/petry.pdf
**Brief behavioral intervention**	Assessment of alcohol use and personalized feedback. Focus on providing a menu of strategies for change, goal setting, empathy, and enhancing self-efficacy.	*General* : Saitz, R. and Galanter, M. (Eds.). *Alcohol/Drug Screening and Brief Intervention: Advances in Evidence-Based Practice.* Binghamton, NY: Haworth Medical Press, 2007. *College students:* Dimeff, L.A.; Baer, J.S.; Kivlahan, D.R.; and Marlate, G.A. *Brief Alcohol Screening and Intervention for College Students (BASICS): A Harm Reduction Approach*. New York: Guilford Press, 1999. *Adolescents:* Monti, P.M.; Colby, S.M.; and O’Leary, T.A. (Eds.). *Adolescents, Alcohol and Substance Abuse: Reaching Teens through Brief Interventions.* New York: Guilford Press, 2001.
**Behavioral couples/family therapies**	Evaluation and treatment of relationship factors that contribute to alcohol use and a focus on increasing relationship factors conducive to abstinence. Incorporates positive activities, communication skills training, and identification of potential relapse triggers.	McCrady, B.S., and Epstein, E.E. *Overcoming Alcohol Problems: A Couples Focused Program. Therapist Guide.* New York: Oxford University Press, 2009. O’Farrell, T.J., and Fals-Stewart, W. *Behavioral Couples Therapy for Alcoholism and Drug Abuse*. New York: Guilford Press, 2006. O’Farrell, T.J., and Fals-Stewart, W. *Behavioral Couples Therapy for Alcoholism and Drug Abuse,* 2002. Available at: http://www.bhrm.org/guidelines/couples%20therapy.pdf
**Facilitated self-change**	Assessment and feedback, motivation information and self-help materials focused on goal-setting, problem solving skills, and self-monitoring.	Hester, R.K., and Miller, W.R. Behavioral self-control training. In Hester, R.K., and Miller, W.R. Eds. *Handbook of Alcoholism Treatment Approaches.* New York: Pergamon Press, 1989. Klingemann, H., and Sobell, L.C., (Eds.). *Promoting Self-Change From Addictive Behaviors: Practical Implications for Policy, Prevention, and Treatment.* New York: Springer Science + Business Media, LLC, 2007. Sobell, M.B., and Sobell, L.C. *Problem Drinkers: Guided Self-Change Treatment.* New York: Guilford Press, 1993.
**Aversion therapy**	Pairing alcohol (sight, taste, or other cue) with an unpleasant experience (including nausea-inducing drugs and electric shock). Covert sensitization uses imagery of aversive scenes paired with imagery of drinking alcohol.	No empirically supported manuals available.

## Footnotes

1 Thus, disulfiram is not technically a pharmacological treatment for alcohol dependence because it only has aversive conditioning properties and does not directly influence alcohol consumption. In contrast, newer medications for alcohol dependence, including naltrexone and acamprosate, have very different mechanisms of action and can reduce alcohol consumption with or without concurrent behavioral treatment (COMBINE Study Research Group 2006).

2 In this study, bona fide psychological treatment was defined as a treatment “that was intended to be fully therapeutic” (Imel et al. 2008, p. 533).

3 The COMBINE Study was a multisite randomized clinical trial designed to test the effectiveness of pharmacotherapy with or without combined behavioral intervention in the treatment of alcohol dependence.
